# Perceptions of fairness in gender relations: a qualitative study of South Korean middle-aged men

**DOI:** 10.1080/17482631.2026.2637803

**Published:** 2026-02-27

**Authors:** Youbin Lee, Soyoung Park, Jueun Kim, Eunha Kim

**Affiliations:** a Department of Psychology, Ajou University, Suwon, South Korea

**Keywords:** Reverse discrimination, Korean men, masculinity, middle-aged men, perceived reverse discrimination, gender equality

## Abstract

**Purpose:**

This study aims to explore how middle-aged men perceive gender equality and fairness, and to investigate the underlying causes and behavioral responses to what they perceive as reverse discrimination. Specifically, it seeks to deeply understand the unique challenges and shifting perceptions of middle-aged men in the context of rapid socio-cultural transitions currently occurring in South Korea.

**Methods:**

This study employed qualitative research methods, conducting semi-structured in-depth interviews with 15 middle-aged men who have children. The collected interview data were rigorously analyzed using the Consensual Qualitative Research (CQR) method to ensure objectivity. The research team individually reviewed the interview transcripts and systematically analyzed the data through three distinct stages: domain identification, core ideas extraction, and cross-analysis.

**Results:**

The study identified four main domains as the causes of perceived reverse discrimination among middle-aged men: uncomfortable expectations and prejudices, perception of preferential treatment towards women, changing roles and responsibilities, and lack of institutional support. Additionally, their responses to perceived reverse discrimination were categorized into three domains: denial/suppression, involuntary compliance, and cognitive restructuring.

**Conclusions:**

This study provides important insights into how middle-aged men react to gender equality policies and broader social changes. These findings offer a foundational basis for psychological counseling and policy development. Furthermore, the results contribute to reducing social conflicts and fostering mutual understanding in the ongoing process of promoting gender equality within Korean society.

## Introduction

South Korean society has long been influenced by Confucian values that emphasise patriarchal family structure and distinct gender roles (Yeo et al., 2017). However, rapid economic growth, globalisation, and expanded educational opportunities for women have led to significant changes in gender relations and to the expansion of policies promoting gender equality (Kim & Park, 2018). In recent decades, the South Korean government has introduced various initiatives, such as support for female entrepreneurship, parental leave policies, and employment subsidies for women returning to the workforce, which have increased women’s participation across multiple social domains.

These developments have been accompanied by growing resistance among some men, who perceive gender equality initiatives as producing unfair disadvantages for themselves (Lee, 2022). This perception, often referred to as perceived reverse discrimination, denotes the subjective belief that policies designed to support historically marginalised groups generate new forms of inequity for majority groups (Sher, 1979). Importantly, the concept of reverse discrimination is politically and empirically contested. Prior research suggests that such perceptions often reflect discomfort with shifting power relations rather than objective or structural disadvantage (Norton & Sommers, 2011; Wilkins & Kaiser, 2014). When established privileges are challenged, these changes may be experienced as discrimination even in contexts where broader structural inequalities persist (Bobo, 2011). Throughout this manuscript, the term ‘reverse discrimination’ is retained because it reflects participants’ own language and terminology used in prior scholarship; however, it is used to denote subjective perceptions and does not imply the existence of structural discrimination against men.

In South Korea, such perceptions are particularly salient among men who argue that sexism is no longer a significant societal issue (Kim, 2017). Survey data indicate that a substantial proportion of men believe contemporary Korean society is biased against men in domains such as employment, intimate relationships, and legal institutions (Chun & Jung, 2019). However, these views must be situated within the context of enduring gender inequality. South Korea continues to rank among the lowest among OECD countries in gender equality, with pronounced disparities in wages, leadership representation, unpaid care labour, and exposure to gender based violence persisting despite policy efforts (Hicks et al., 2025; OECD, 2024).

Against this background, the present study was aimed to examine how middle-aged South Korean men perceive and interpret what they describe as reverse discrimination within a context of ongoing gender inequality and social change. Rather than treating these perceptions as evidence of structural discrimination against men or attempting to determine whether such discrimination exists, we approached them as a social and psychological phenomenon shaped by men’s experiences of changing expectations, responsibilities, and social roles. This framing allows for careful analysis of subjective experiences while maintaining critical distance from claims of objective disadvantage.

Empirical research on perceived reverse discrimination among South Korean men remains limited. Nevertheless, insights can be drawn from broader literature on perceived discrimination and intergroup threat. Previous studies have shown that perceived discrimination is associated with negative psychological outcomes, including psychological distress, depression, and anxiety (Cheref et al., 2019; Pascoe & Richman, 2009), with effects that may persist over time. Research focusing on South Korean men has further linked perceptions of reverse discrimination to feelings of unfairness, resentment, and reduced organisational commitment (Kim et al., 2022; 2021; Lee et al., 2022). However, it remains unclear whether these perceptions translate into objective disadvantages or instead reflect psychological responses to changing social hierarchies.

In light of these limitations in existing research, the present study focuses on middle-aged men for several reasons. This cohort represents a transitional generation that bridges traditional patriarchal values and emerging norms of gender equality (Lee & Kim, 2014). As a result, middle-aged men may experience tension between longstanding expectations related to breadwinning and masculine authority and newer demands for shared domestic labour and more egalitarian partnerships, which can give rise to perceptions of reverse discrimination in South Korea’s rapidly changing society. In addition, middle-aged men continue to occupy positions of relative power in South Korean social and institutional contexts, making their attitudes toward gender equality particularly consequential. Examining their perceptions therefore provides insight into both individual experiences and broader social dynamics.

### Causes of perceived reverse discrimination in South Korea

Men’s perceptions of reverse discrimination can be understood as responses to perceived changes in social status and gender relations. Research on intergroup relations suggests that when members of historically dominant groups perceive threats to their social standing, they may adopt defensive interpretations to protect their perceived position (Blodorn et al., 2012). Scholarship on masculinity further indicates that resistance to gender equality is often linked to anxiety about changing gender roles, particularly among men who strongly endorse traditional masculine ideals (Borinca et al., 2021). Together, these perspectives provide a framework for examining how men interpret gender equality initiatives in contexts of rapid social change.

In South Korea, these dynamics have unfolded alongside visible institutional changes related to gender equality. Two developments have been especially influential: the abolition of the military bonus point system and the introduction of gender quota policies. The military bonus system, which granted advantages to men in public sector employment following mandatory military service, functioned as an institutional privilege that excluded women. Its abolition in 1999 was interpreted by some men not as the removal of unequal advantage, but as reverse discrimination (Ahn, 2007). Similarly, gender quota policies have often been perceived as preferential treatment, despite their intent to address women’s persistent underrepresentation in leadership and decision-making roles (Lee, 2013).

Broader economic and social conditions further shape these perceptions. Prolonged economic stagnation and intensified competition in the labour market have heightened concerns about job security and career mobility among middle-aged men (Song, 2021). In this context, some men are more likely to interpret women’s advancement as occurring at their expense. At the same time, middle-aged men often experience tension between expectations related to breadwinning and authority and growing demands for participation in domestic labour and more egalitarian partnerships. This tension can produce role strain and uncertainty about masculine identity (Kim & Hwang, 2019).

Finally, changes in media representation and public discourse have contributed to how these perceptions are reinforced and shared. The increased visibility of feminist movements, including #MeToo, has led some men to feel that they are being unfairly associated with broader gender related problems (Kim et al., 2022). Such sentiments are often amplified in online spaces where individual frustrations are reframed as collective experiences of unfair treatment (Jung, 2023). These reactions are better understood as responses to social and cultural transition rather than as evidence of systemic discrimination against men.

#### Purpose of this study

While research on gender discrimination against women in South Korea has been extensive, middle-aged men’s perceptions of reverse discrimination remain underexplored. This study examines these perceptions using a critical qualitative approach that situates men’s subjective experiences within the broader context of persistent gender inequality. Specifically, we explore the situations in which middle-aged men perceive unfair treatment, how they interpret and respond to these experiences, and how such perceptions are embedded in wider gender relations.

Examining these perceptions is important for several reasons. First, middle-aged men continue to exert substantial social and economic influence in South Korea, shaping workplace practices, family norms, and public discourse. Understanding how this group interprets gender equality initiatives therefore provides insight into resistance to social change and the negotiation of shifting gender hierarchies. Second, the South Korean context is particularly salient given its paradoxical character: despite ranking among the most gender unequal countries in the OECD, public discourse increasingly frames gender relations as a zero-sum conflict in which men are portrayed as disadvantaged (Jung, 2023). By examining when and how perceptions of reverse discrimination arise, and by critically assessing whether they reflect structural disadvantage or discomfort with changing social hierarchies, this study seeks to clarify the psychological and social processes underlying contemporary gender tensions.

#### Theoretical framework

This study draws primarily on Connell’s theory of hegemonic masculinity, supplemented by recent scholarship on masculinity and social change. Hegemonic masculinity refers to socially privileged forms of masculinity that emphasise authority, heterosexuality, and emotional restraint, while marginalising alternative masculinities and reinforcing gender hierarchies (Connell & Messerschmidt, 2005). When gender equality initiatives challenge these hierarchies, men who have internalised hegemonic masculine norms may experience threats to their gender identity, which can lead to defensive interpretations expressed as perceptions of reverse discrimination.

Empirical research supports this interpretation while also highlighting variation in men’s responses to social change. Men who strongly endorse traditional masculinity norms report heightened discomfort when gender roles are perceived to be changing (Borinca et al., 2021). Borinca and Gkinopoulos (2025) further show that heterosexual men tend to endorse traditional masculinity more strongly and report lower emotional vulnerability than sexual minority men, partly because they are less likely to embrace nontraditional domestic and relational roles. Importantly, exposure to alternative masculine role narratives was found to reduce rigid masculinity endorsement and increase emotional openness. These findings indicate that men’s responses to gender equality initiatives vary depending on how central traditional masculinity is to their identity. This variation helps explain why some middle-aged South Korean men adapt more flexibly to changing gender norms, while others respond more defensively.

Recent scholarship conceptualises perceptions of reverse discrimination as part of masculinity contestation rather than simple opposition to gender equality. In this process, men may endorse egalitarian ideals in principle while expressing resentment or discomfort toward specific policies or cultural changes that they perceive as disadvantaging them. This pattern parallels research on ambivalent sexism, which highlights the coexistence of egalitarian beliefs with defensive or paternalistic responses during periods of normative change (Glick & Fiske, 1996). From this perspective, perceptions of reverse discrimination reflect ongoing negotiations over masculinity within changing social and institutional contexts.

An intersectional perspective further underscores that men’s engagement with hegemonic masculinity is not uniform across social positions (Crenshaw, 1989). Socioeconomic conditions, employment security, education, and regional context may shape how gender equality initiatives are interpreted and emotionally experienced. For example, men facing job insecurity or limited upward mobility may be more likely to interpret women’s policy gains as direct threats to their own economic stability, whereas men with more secure occupational positions may experience the same changes as less destabilising. Although the present study does not permit systematic examination of these intersecting dimensions due to the relative homogeneity of the sample, integrating hegemonic masculinity theory with an intersectional lens situates men’s subjective perceptions within broader structural dynamics. This approach allows for a critical examination of whether perceptions of reverse discrimination reflect structural disadvantage or discomfort with the erosion of historically privileged positions.

## Methods

### Participants

Fifteen middle-aged South Korean men participated in this study. The eligibility criteria were as follows: age between 40 and 59 years, identified as cisgender male, married, and having at least one child. After receiving approval from our institution's IRB(#202401-HS-001), we recruited participants through advertisements on platforms such as NaverCafé, Facebook, Instagram, and community newsletters. Eligibility was assessed through email or phone screening. Eligible participants were scheduled for a phone interview after being informed of the study's purpose, procedure, voluntary nature of participation, and confidentiality. Written informed consent was obtained via email before the interviews. Each participant received $25 compensation. Participants’ ages ranged from 40 to 57 years (*M* = 43.53, *SD* = 5.08). Regarding occupation, 66.6% (*n* = 10) held clerical roles, 20% (*n* = 3) held managerial positions, 6.7% (*n* = 1) held professional occupations, and 6.7% (*n* = 1) held production roles.

### Procedure

We developed an interview protocol based on a review of qualitative studies on reverse discrimination and inputs from middle-aged men. The protocol consisted of four primary contexts: (1) family relationships (e.g., domestic labour, parenting), (2) workplace settings (e.g., hiring, promotion), (3) social systems and laws (e.g., government policies, military service), and (4) other social situations (e.g., public spaces, interpersonal interactions). Interviews began with a general question about experiences of reverse discrimination as men and concluded with an open-ended question inviting participants to share additional experiences not covered earlier. Follow-up probes were used throughout to elicit detailed accounts and clarify responses.

The interview protocol was pilot tested with two middle-aged men who were postdoctoral fellows in a psychology department. Based on their feedback, questions were refined for clarity and reordered as needed. All interviews were conducted in Korean via telephone, audio-recorded with participants’ consent, and transcribed verbatim by the research team. Quotations used in the manuscript were translated into English by one author and reviewed for accuracy by an external auditor.

### Research team

The research team consisted of a postdoctoral research fellow, a doctoral student, and a master’s student, all affiliated with a psychology department in South Korea. All team members were cisgender women with prior experience in qualitative research on gender and discrimination. To enhance methodological rigour, three external auditors, all male psychologists with experience in Consensual Qualitative Research, provided feedback during the analytic process. Before data analysis, the research team reviewed the methodological guidelines outlined by Hill et al. (2005) to ensure a shared understanding of CQR procedures and analytic principles.

Given that all members of the research team were women, we explicitly acknowledged the potential influence of gender-related assumptions in interpreting men’s accounts of perceived reverse discrimination. To address this, the team engaged in a reflexive analytic process that involved documenting assumptions, engaging in regular discussions to challenge interpretations, and grounding analytic decisions in participants’ own language. External male auditors reviewed preliminary codes and thematic structures after the initial coding phase and provided interpretive feedback rather than simple verification.

The auditors’ input was particularly important in refining thematic interpretations. For example, when participants described increased burdens related to childcare or housework, auditors encouraged the research team to consider these accounts as reflecting unfamiliarity with roles historically assigned to women rather than resistance to gender equality. Similarly, when themes such as perceived favouritism toward women or being treated as a suspected perpetrator emerged, auditors emphasised the importance of distinguishing between subjective experiences of unfairness and claims of structural disadvantage. These discussions prompted revisions to theme labels and repeated returns to the original transcripts to ensure that interpretations remained closely aligned with participants’ intended meanings.

### Data analysis

To analyse our data, we used CQR (Hill et al., 2005) to analyse our data, a method that is particularly effective for exploring hidden or underexplored topics that lack standardised measurement tools. Due to the limited literature on South Korean middle-aged men's perceptions of reverse discrimination, the CQR was chosen for this study. Additionally, CQR's collaborative team approach, in which multiple researchers analyse data together, helps reduce individual biases and includes diverse perspectives (Hill et al., 2005). The process involved three steps: developing domains, extracting core ideas, and conducting a cross-analysis.

In the first phase, team members individually reviewed the interview transcripts and identified preliminary domains. They then met to reach consensus on these domains. Following the completion of the initial coding, external auditors evaluated and provided feedback. The feedback was discussed and integrated into the coding framework, such as changing “societal expectations and pressures” to “societal expectations regarding success and achievement” and adding “policies that give women an unfair advantage.”

During the second phase, the team members individually developed core ideas that captured the essence of the participants' statements within each domain. Through consensus-building discussions, the team refined the core ideas. Once agreed upon, the auditors reviewed them and suggested minor revisions such as reassigning certain core ideas to different domains and revising the names of domains. For example, in the domain of “Perceived favouritism for women,” male auditors noted that the initial coding did not fully capture participants' nuanced perspectives on workplace dynamics. Specifically, the subcategory “Policies that grant women an unjust advantage” was initially labelled more neutrally as “Perceptions of workplace policies.” Male auditors emphasised that the participants’ language often conveyed frustration or resentment about such policies, which prompted us to adjust the subcategory to reflect the emotional undertones of the participants' narratives more accurately. Similarly, in the domain “Uncomfortable Expectations and Biases,” male auditors provided insights that clarified the subcategory “Men's voices often being disregarded.” While the research team initially interpreted this as a general perception of unfair treatment, male auditors highlighted that participants frequently tied this sentiment to specific interactions, such as being overlooked during family decision-making or in workplace settings. This feedback refined the coding to reflect the context in which the participants felt that their voices were marginalised.

Each team member then individually conducted a cross-analysis, developing categories that captured the core ideas within each domain. The team reviewed the transcripts to ensure core ideas within categories were accurate and made revisions as needed, like separating “men's voices often being disregarded” and “gender double standards.” In accordance with Hill et al.'s (2005) coding standard, categories were classified as “general” for 14-15 cases, “typical” for 8–13 cases, and “variant” for 3–7 cases. It should be noted that with a sample size of fifteen, variant categories represent 20-47% of the participants, warranting substantive consideration. Exceptional cases with only 1-2 instances were excluded from analysis. These frequency labels indicate the relative consistency of thematic patterns across cases within this specific sample and are not intended to imply statistical prevalence or generalisability beyond the study participants.

To ensure the accuracy and validity of our findings, external male auditors reviewed the finalised domains and categories. This final review confirmed that our coding framework aligned with the participants’ experiences. For instance, when disagreements arose about the interpretation of “Valuing and protecting women” in the domain “Perceived Favouritism for Women,” male auditors noted that participants’ statements often conveyed mixed feelings of both appreciation and frustration. This feedback enabled us to refine the subcategories to capture these ambivalent emotions more accurately.

## Results

Data analysis using the CQR revealed seven domains and 17 categories. Tables I and II summarise the findings. The domains and categories are described below, and quotes from participants illustrate the main findings.

**Table I.** Causes of perceived reverse discrimination reported by the participants.

Domains	Categories	Frequency
Uncomfortable expectations and biases	Societal expectations for success and achievement	General
	Suspected perpetrator	Typical
	Men's voices often being disregarded	Typical
	Gender double standards	Variant
Perceived favouritism for women	Women's perception of gender equality in their favour	Typical
	Policies that grant women an unjust advantage	Typical
	Valuing and protecting women	Typical
	Assigning challenging tasks to men	Variant
Changing roles and responsibilities	Increased burden of childcare and housework	Typical
	Having to navigating between older and younger generations	Typical
Lack of institutional support	Discrepancy in the use of parental leave by men and women	Typical
	Lack of compensation for military service	Typical

### Causes of perceived reverse discrimination

Four domains and twelve categories emerged for the first research question. These domains and categories are related to the factors or reasons behind instances in which the participants perceived that they had been subjected to perceived reverse discrimination.

#### Uncomfortable expectations and biases

This domain focuses on four categories related to perceived reverse discrimination among men due to uncomfortable or burdensome expectations and biases. All participants, with the exception of one, talked about being frequently pressured to have high social status and economic power simply because they are middle-aged men (“societal expectations regarding success and achievement”). While these expectations are not always negative, they can be distressing for those who feel that they are not meeting them. One participant noted, “I'm the only man among all my female coworkers.

Visitors to our office often assume that I must be my boss. They expected a middle-aged man such as me to hold a higher level of authority or position. Such encounters leave me feeling quite bewildered.” (#3)

Eleven participants expressed concerns about being assumed potential perpetrators of sexual harassment and violence in the workplace or other social settings. One participant shared, “I was once accused of sexual harassment by a female colleague. Instead of upholding a presumption of innocence, I encountered a presumption of guilt. Despite the lack of evidence, the organisation pressured me to prove my innocence. I was repeatedly coerced into draughting statements and later asked to write a statement of remorse.” (#1) Another participant pointed out, “If you look at the illustrations in sexual harassment prevention education, the perpetrator is depicted as a man and the victim as a woman. I have never seen the perpetrator to be a woman. This alone suggests that men are inherently perpetrators and implies that men are criminals.” (#4)

Furthermore, several participants mentioned that their opinions were not valued because women held more decision-making power (with “men's voices often being disregarded”). Examples include, “Nowadays, there’s almost nothing that a man can decide, especially at home. I must follow my wife’s opinions on most matters. After 20 years of living like this, her opinion is the law at home” (#13) and “A man in a family is usually the one who is below the dog in the hierarchy. I am not that different. It would have been nice if we [my wife and I] were equal.” (#2) Recalling these experiences, the four participants discussed how men and women are judged differently for the same behaviour (referred to as the “gender double standard”). For instance, participants noted, “Men can get sick, just like women can, but it's hard for men to admit they're sick. When they do, they're told to suck it up and not to exaggerate” (#14) and “A female boss touching a male employee's shoulder is seen as encouragement, while a male boss doing the same to a female employee is considered very inappropriate.” (#3)

#### Perceived favouritism for women

This domain includes four categories related to how men view favouritism toward women as a form of reverse discrimination. The first category (women’s interpretation of gender equality in favour of themselves), identified by 11 participants, involved women advocating for gender equality while expecting men to adhere to traditional gender roles. For example, one participant mentioned, “My wife pushes for what she wants, emphasising that this is for the purpose of achieving gender equality. However, at the same time, she requests that I assume the role of the sole breadwinner.” (#6) Another participant shared, “When we got married, my wife and I made a commitment to each other to work hard and save money together. However, as time passed, she assumed that I should contribute more to living expenses and the purchase of expensive items.” (#8) These accounts reflect tensions in negotiating changing gender expectations within intimate relationships, rather than clear evidence of structural advantage for women. They illustrate how participants interpreted shifts in domestic and economic roles through the lens of perceived inconsistency in gender norms.

Furthermore, in the second category (“policies that give women an unfair advantage”), nine participants noted that specific policies designed to promote gender equality, such as designated parking spots for women and reduced physical standards for women during the hiring process, may lead to reverse discrimination against men. For example, one participant stated, “In the past, lowering physical standards in the hiring process of professions such as police, military, or firefighting was necessary for women who were socially disadvantaged. However, women no longer require these advantages. They are ranked as top candidates for large companies and civil service examinations. The practice of preferential physical testing of women is unfair to men.” (#8) Similarly, another participant observed, “A women-only break room was available at work, but there was no men-only break room.” (#5) These interpretations should be understood within the broader context of persistent structural gender inequality in South Korea, where such measures were introduced to address documented underrepresentation and wage disparities (OECD, 2024). Framing them as unfair advantages reflects participants’ perceptions rather than objective evidence of reversed hierarchy.

In addition, the third category (“having to value and protect women”) revealed that eight participants experienced reverse discrimination by prioritising women's needs to show value and protection. For instance, one participant mentioned, “Because women tend to be more sensitive than men, I usually ask my female colleagues about their needs when I plan a company dinner and workshop.” (#13) Another participant stated, “I try to give women less challenging, low-risk projects as a gesture of consideration, which can be unfair to men who have to take on high-stress tasks.” (#2) Furthermore, this category intersects with the fourth category (“assigning difficult tasks to men”), with five participants sharing instances where men were consistently given more challenging assignments. Some comments included, “I think the Korean workplace itself has a bit of an atmosphere where women are not given difficult and important tasks. I rarely see women taking on difficult tasks or doing difficult tasks themselves. If there is something new or difficult, men are usually called” (#1) and “If you must work nights or weekends, it’s usually the men who do it. It is the same as company dinners, where most women do not go or leave earlier, but men must go even if they do not want to do so.” (#7)

#### Changing roles and responsibilities

This domain includes two categories concerning how men's changing roles and responsibilities can lead to reverse discrimination. The first category, “Increased burden of childcare and housework,” refers to the growing expectations for men to handle childcare and housework duties, even in single-income households. Participants recognised that becoming more involved in these areas could have benefits, such as forming stronger bonds with their children and fostering gender equality at home. However, they also shared feelings of stress and exhaustion from juggling family related tasks and work obligations. One participant mentioned, “Although I appreciate spending more time with my children, balancing childcare and my job is incredibly exhausting.”. (#5)

The second category, “having to navigate between older and younger generations,” highlights the challenge of balancing the expectations of both age groups. According to the participants, older generations value loyalty, long working hours, and hierarchical structures, while younger generations prioritise work-life balance, flexibility, and a flat organisational structure. Participants noted that, as a “sandwich generation,” they often feel caught between adhering to traditional expectations from older colleagues and adapting to progressive values from younger colleagues. This balancing can cause stress and a sense of being unfairly targeted or misunderstood, particularly when criticism is received from both sides. Moreover, participants noted that they felt unfairly assigned the role of mediator, as this responsibility was not shared by their female counterparts. One participant stated, “In the past, we did whatever the boss said. However, to get young people into work, they must obtain their consent and explain the benefits of the task. Bosses do not understand this young generation and get angry at me for not getting things done. I have to satisfy the demands of the older generation while being careful of the younger generation.” (# 12)

#### Lack of institutional support

This domain includes two categories related to institutional-level perceptions of reverse discrimination. The first category, “Discrepancy in the use of parental leave between men and women,” reflects participants’ views that paternal leave remains socially discouraged and economically risky. Although maternity leave is widely accepted, participants reported that men often avoid taking leave due to concerns about promotion disadvantages, negative workplace evaluations, and income loss. As one participant stated, “When men take paternity leave, most people react negatively and say, ‘Did you give birth? Are you sick? What’s wrong with you?’ Even women would say, ‘It’s a woman who gave birth, so why should a man take a break?’” (#3) Participants also noted that in households where men serve as primary breadwinners, financial pressures further deter leave-taking, sometimes with spouses opposing paternal leave due to concerns about economic stability.

The second category, “Lack of compensation for military service,” highlights the absence of compensation for mandatory military service, which lasts for a minimum of 18 months. Concerns were raised by the participants regarding the lack of compensation for men in the military, which created an unfair competitive environment for women. One participant said, “I think it's a shame that the military bonus has been eliminated because it is unfair that men have to compete with women after spending a year and a half in the military without any bonus. This is an example of reverse discrimination.” (# 5) Another participant agreed, “I agree that men should serve in the military because it is a legal duty. However, men deserve extra points to fulfil this duty.” (#14)

### Responses to perceived reverse discrimination

The participants’ responses to perceived reverse discrimination for the second research question were grouped into three domains and five categories. These categories represent the various reactions that individuals display when experiencing reverse discrimination (see Table II).

**Table II.** Participants' responses to experiences of perceived reverse discrimination.

Domains	Categories	Frequency
Denial/Suppression	Being hesitant to admit having experienced perceived reverse discrimination	Typical
	Suppressing emotions	Typical
Involuntary compliance	Avoiding conflicts and confrontations	Typical
	Sacrificing one's own needs	Typical
Cognitive reframing	Viewing perceived reverse discrimination as a transitional phenomenon	Variant

General: 14-15 cases, typical: 6–13 cases, variant: 2–5 cases.

#### Denial/Suppression

This domain examines how men ignore, minimise, or dismiss their perceptions of reverse discrimination. The first category, “Being hesitant to express having experienced reverse discrimination,” refers to the participants’ reluctance to openly acknowledge or discuss instances of reverse discrimination they encountered. Examples include: “It’s hard to call it reverse discrimination. There’s this lingering doubt that if it’s just me overreacting or if it’s a real issue (#13)” and “There are still high levels of discrimination against women. So, it is hard to talk about instances when you are discriminated against as a man.” (#10) Additionally, the second category, “suppressing emotional expression”, indicates that participants reported withholding emotional responses related to experiences of reverse discrimination. For example, “When I feel hurt by unfair treatment, I either put up with it or just kind of try to deal with it independently. Or I withdraw from the outside world until I feel better.” (#11) and “It’s because I’ve been taught that way since I was kind, so I’m a little bit embarrassed to express my feelings. It’s a bit even shameful.” (#15)

#### Involuntary compliance

This domain captures participants’ tendency to respond to perceived reverse discrimination by complying rather than confronting the situation. The first category, “Avoiding confrontation,” reflects efforts to maintain harmony in workplace and family settings by remaining silent or refraining from challenging perceived unfairness. As one participant explained, “When I feel I am being treated unfairly at work, I choose to stay quiet and avoid escalating the situation. I do not want to create conflict, so I just let it go.” (#5)

The second category, “Sacrificing one’s own needs,” describes instances in which participants accepted additional workloads or unfavourable conditions to avoid being seen as disruptive. For example, one participant stated, “I often agree to extra work because I do not want to be viewed as uncooperative. It is easier to go along with it than to fight back.” (#6)

#### Cognitive reframing

This domain includes one category (“viewing reverse discrimination as a transitional phenomenon”). This highlights how men view reverse discrimination as a temporary stage on the path to achieving gender equality. Participants believed that their current experiences of reverse discrimination were necessary for the evolution of gender roles. They understand that changes promoting gender equality may temporarily put men at a disadvantage, but see these changes as leading to a more balanced and fair society in the long run. For example, one participant said,’ Seeing the bigger picture helps me cope with reverse discrimination. To achieve long-term gender equality, there may be periods during which men feel disadvantaged. It is part of the growing pain of transitioning to a society where everyone has equal opportunities, regardless of gender.” (#9)

## Discussion

This study employed the CQR method to examine sources of perceived reverse discrimination and how a small, relatively homogeneous group of middle-aged South Korean men described and interpreted these experiences. Given the characteristics of the sample (married, cisgender, employed fathers), the findings presented below should be understood as reflecting this specific subgroup rather than middle-aged South Korean men more broadly. In this section, we interpreted the main findings in relation to prior research, situated them within the broader gender context of South Korea, and discussed implications for counselling and policy, while acknowledging the study’s limitations.

### Major findings and theoretical interpretation

The findings indicate that middle-aged South Korean men’s perceptions of reverse discrimination arise from several recurring experiences reported in the interviews, including discomfort with changing gender expectations, perceptions of preferential treatment toward women, increased involvement in domestic responsibilities, and a perceived lack of institutional support. Similar patterns have been reported in prior research conducted in societies undergoing shifts in gender relations, where members of historically advantaged groups interpret equality-oriented changes as personally disadvantageous (Norton & Sommers, 2011; Wilkins et al., 2015).

Participants’ discomfort with being viewed as potential perpetrators of sexual harassment does not necessarily imply opposition to gender equality. Instead, it often stems from uncertainty regarding behavioural expectations in a changing social context. Prior research indicates that men accustomed to traditional masculinity norms face difficulties when those norms provide limited guidance for new interpersonal expectations (Borinca et al., 2021). Viewed in this light, participants’ accounts are better understood as responses to shifting social scripts rather than a refusal to acknowledge women’s claims of harm.

In addition, participants’ accounts regarding favouritism toward women illustrate a contradictory attitude toward gender equality. Some participants appeared to endorse traditional male privileges, such as expecting deference from women, while expressing reluctance to share traditional male obligations. This pattern aligns with ambivalent sexism theory (Glick & Fiske, 1996), which describes how egalitarian and traditional views can intertwine to maintain gender hierarchies. From this perspective, perceptions of reverse discrimination may represent an attempt to reconcile changing norms with traditional status, rather than indicating actual structural disadvantage.

Similarly, participants’ perception that gender equality policies constitute unfair advantages must be understood against the backdrop of persistent structural inequality. Given that women remain significantly underrepresented in leadership and continue to experience substantial wage gaps in South Korea (OECD, 2024), the policies in question were implemented to address these specific barriers. Viewing such measures as reverse discrimination reflects zero-sum thinking (Kuchynka et al., 2018; Norton & Sommers, 2011), where gains for women are perceived as losses for men.

Furthermore, participants’ claims of increased domestic burdens must be critically examined in light of South Korea’s pronounced gender imbalance in unpaid labour. Despite reported shifts in behaviour, objective data indicate that men continue to perform substantially less childcare and housework than women (Craig & Mullan, 2010; Hong & Lee, 2012). This discrepancy underscores the necessity of distinguishing between men’s subjective experiences of role strain and the objective reality of structural inequality, and challenges the validity of their claims regarding reversed gender roles.

The response patterns identified—denial or suppression, reluctant compliance, and cognitive reframing—are consistent with prior research on emotional expression among middle-aged Korean men, which emphasises restraint, self-control, and avoidance of open conflict (Kwon et al., 2013; Lee et al., 2014). Rather than indicating indifference, these strategies suggest efforts to manage discomfort privately in situations perceived as politically sensitive or socially contentious.

### Limitations

These findings should be interpreted in light of several methodological and conceptual limitations. First, while we collected basic demographic information, we did not examine how factors such as socioeconomic status, education level, employment status, sexual orientation, or regional background might shape perceptions of reverse discrimination. This homogeneity limits generalisability and precludes a fully intersectional analysis. Future research should include more diverse samples to better capture these intersecting influences on men’s responses to gender equality initiatives.

Second, recruitment through online platforms may have introduced selection bias. Participants who responded to online advertisements were likely more engaged with gender-conflict discourse or more motivated to articulate grievances related to gender issues than the broader population of middle-aged men. As a result, the sample may reflect perspectives that are more salient or strongly articulated than those found in the general population.

Third, the gender composition of both the interviewers and the research team may have influenced data generation and interpretation. All interviews were conducted by cisgender women via telephone, which may have affected the depth and openness of participants’ disclosures on sensitive topics related to gender and masculinity. The telephone format also limited access to nonverbal cues and may have constrained rapport building. Prior research suggests that middle-aged men tend to exhibit relatively low levels of emotional self-disclosure in such contexts (Lee & Kim, 2024), and this tendency may have been further shaped by the gender dynamics between female interviewers and male participants, as well as by the absence of nonverbal cues inherent in telephone interviews.

In addition, although steps were taken to address potential gender-related bias during analysis through reflexive discussions, the involvement of male external auditors, and grounded coding practices, the all-female composition of the core research team may still have shaped interpretive decisions. Some nuances of men’s experiences may therefore have been emphasised or interpreted differently than they might have been within a more gender-diverse analytic team. Future research would benefit from incorporating male interviewers, mixed-gender research teams, or extended rapport-building prior to addressing sensitive gender-related topics.

### Implications

Despite these limitations, this study offers insight into how middle-aged South Korean men interpret and respond to gender equality initiatives in everyday contexts. The findings have implications for both clinical practice and policy, particularly in addressing men’s subjective experiences while remaining attentive to persistent gender inequalities.

### Clinical implications

When working with South Korean middle-aged men in individual therapy, clinicians should actively explore the clients’ perceptions of reverse discrimination. Given their tendency to exhibit denial/suppression and involuntary compliance, clinicians must create a safe, nonjudgmental space for open discussion. Validating clients’ experiences while simultaneously fostering critical reflection is also important. For example, clinicians can use Socratic questioning or guided exploration techniques to help clients examine how traditional gender norms and systemic gender inequities contribute to their perceptions. This approach allows clients to process their emotions while gaining a broader understanding of societal contexts.

Additionally, clinicians should examine how traditional gender roles and stereotypes, such as expectations of strength and success, negatively impact middle-aged South Korean men. In addition, clinicians can use cognitive restructuring techniques to help clients challenge rigid beliefs about masculinity and reframe masculinity by identifying and fostering healthy aspects of male gender roles such as responsibility, courage, perseverance, and resilience (O’Neil, 2008). Furthermore, clinicians should encourage male clients to explore situations where they perceive favouritism towards women and engage in open conversations with their spouses and female colleagues to prevent misunderstandings. Addressing benevolent sexism and its consequences can also help clients recognise the impact of these perceptions on women's abilities and opportunities (Becker & Wright, 2011).

Finally, South Korean middle-aged men would benefit from strategies to manage the dual burden of being primary breadwinners and having increased responsibilities for childcare and housework, as well as navigating intergenerational expectations. For instance, clinicians can assist clients in developing time management and problem-solving skills to navigate these demands effectively. Encouraging clients to seek or establish support systems such as community groups for fathers or workplace peer networks, can also provide emotional and practical support. Additionally, integrating mindfulness-based stress reduction (MBSR) or relaxation training into therapy can address stress related to their dual roles.

#### Policy implications

The findings suggest that policy responses should take men’s perceptions of unfairness seriously without framing them as evidence of structural disadvantage. Participants frequently described feeling overburdened by work and family responsibilities in the context of changing gender roles. Policies that promote men’s use of parental leave, such as stronger job protection and clearer signals that leave-taking does not entail career penalty, may reduce these concerns while supporting more equitable caregiving arrangements.

Participants also raised concerns related to military service and transitions into civilian employment. Practical support for skill development and career planning during and after military service may help address these anxieties as part of broader workforce support, rather than as compensation for disadvantage. In addition, workplace and educational initiatives that challenge rigid gendered expectations, such as assumptions about men’s emotional restraint or physical endurance, may ease resistance to gender equality initiatives. Policy approaches are likely to be most effective when they address men’s lived experiences while remaining clearly aligned with efforts to reduce women’s continuing structural inequalities.

## Conclusion

This study examined perceptions of reverse discrimination among middle-aged South Korean men within the context of changing gender relations. The findings indicate that participants often interpreted gender equality policies and shifting norms in terms of personal burden, role expectations, and social responsibility. These interpretations reflect the position of middle-aged men as a transitional generation situated between traditional patriarchal values and emerging egalitarian norms. Importantly, the findings represent subjective perceptions and do not constitute evidence of objective or systemic discrimination against men. Instead, the study illustrates how members of a historically privileged group psychologically and socially respond when established gender hierarchies are challenged.

## Data Availability

The data supporting the findings of this study are available from the corresponding author upon reasonable request, subject to IRB approval.
